# 2114 Severity of childhood-onset systemic lupus erythematosus: Impact of preceding and co-existing autoimmune cytopenias (protocol)

**DOI:** 10.1017/cts.2018.120

**Published:** 2018-11-21

**Authors:** Ekemini Akan, Shanmuganathan Chandrakasan, Kelly Rouster-Stevens, Laurence Greenbaum, Chelsea Marion, Karli Singer, Ignacio Sanz, Sampath Prahalad

**Affiliations:** 1 Emory University; 2 Morehouse School of Medicine

## Abstract

OBJECTIVES/SPECIFIC AIMS: The goals of our study are: (1) To test the hypothesis that the presence of any autoimmune cytopenia (ITP, AIHA, or ES) at time of cSLE diagnosis is associated with decreased risk of developing LN. (1b) To test the hypothesis that there is a lower risk of LN in patients with cSLE and any co-existing autoimmune cytopenia (ITP, AIHA, or ES) who had treatment with immunomodulatory or immunosuppressive therapy (intravenous immunoglobulin, corticosteroids, rituximab, or cyclophosphamide) before diagnosis of cSLE. (2) To test the hypothesis that in patients with cSLE who develop LN, the presence of any co-existing autoimmune cytopenia (ITP, AIHA, or ES) at time of cSLE diagnosis is associated with less severe LN. (3) To test the hypothesis that at the time of cSLE diagnosis, there is a lower incidence of double-stranded DNA (dsDNA) and a higher incidence of ribonucleoprotein autoantibodies in those with co-existing autoimmune cytopenias (ITP, AIHA, or ES). METHODS/STUDY POPULATION: This is a retrospective study of a large cohort of patients from the Emory Children’s Center, Children’s Healthcare of Atlanta (CHOA) satellite clinics and pediatric rheumatology inpatient services at any of the 3 CHOA hospitals (Egleston, Scottish Rite, and Hughes Spalding) with ICD 9 or ICD 10 codes corresponding to a diagnosis of SLE between January 1, 2000 and January 31, 2015. We will include patients diagnosed at age 2–16 years who meet at least 4 of the 11 American College of Rheumatology (ACR) classification criteria for SLE. We will consider these patients as having cSLE. We will exclude patients with less than 2 years of follow-up data and patients with a pre-existing diagnosis of cSLE who transferred care to our Emory/CHOA center. We will define time of diagnosis as time from initial evaluation for cSLE by a pediatric rheumatologist up to 28 days post cSLE diagnosis. We will define co-existing autoimmune cytopenia as preceding diagnosis of a primary autoimmune cytopenia or the presence of an autoimmune cytopenia at the time of initial evaluation for cSLE and up to 28 days post cSLE diagnosis. We will define AIHA as hemoglobin ≤10 g/dL with positive direct Coombs and/or reticulocytosis. We will define ITP as thrombocytopenia <100,000/mm^3^ and Evans syndrome as concurrent or sequential AIHA and ITP. We will define lupus nephritis (LN) as the presence of urine protein to creatinine ratio>0.5 in a patient with cSLE and/or biopsy demonstrating LN. IRB approval of the study protocol with waiver of informed consent has been obtained from the CHOA IRB. RESULTS/ANTICIPATED RESULTS: We have approximately 40 newly diagnosed cSLE patients annually; therefore, a study population of 400 patients with cSLE is possible. Therefore, assuming 50% of cSLE patients without autoimmune cytopenias have LN and 22% of cSLE patients with autoimmune cytopenias have LN, at an alpha of 0.05, we will have > 80% power to detect significant differences. We expect to show phenotypic differences in patients with co-existing autoimmune cytopenia and cSLE from other newly diagnosed cSLE patients. We expect that the presence of a co-existing autoimmune cytopenia and cSLE is associated with decreased risk of developing LN. We expect that there will be a decreased prevalence of LN in cSLE patients pretreated with immunosuppression further highlighting that earlier indicators of LN risk and early interventions are necessary. We expect to find decreased severity of LN in patients with a co-existing autoimmune cytopenia at time of cSLE diagnosis. DISCUSSION/SIGNIFICANCE OF IMPACT: Our study will be conducted on one of the largest single-center cohorts of cSLE patients. We will determine whether pediatric patients with SLE and autoimmune cytopenias have a distinct clinical or serological phenotype and less severe disease. Our results will be significant in developing hypothesis for further retrospective or prospective multi-center or large database and immunological studies to understand the relationship of each individual autoimmune cytopenia to cSLE. It will provide the necessary background for further clinical and immunological studies to identify predictive biomarkers of cSLE severity.

